# Systematic transcriptomic analysis and temporal modelling of human fibroblast senescence

**DOI:** 10.3389/fragi.2024.1448543

**Published:** 2024-08-29

**Authors:** R.-L. Scanlan, L. Pease, H. O’Keefe, A. Martinez-Guimera, L. Rasmussen, J. Wordsworth, D. Shanley

**Affiliations:** ^1^ Campus for Ageing and Vitality, Newcastle University, Newcastle, United Kingdom; ^2^ Center for Healthy Aging, Institute of Cellular and Molecular Medicine, University of Copenhagen, Copenhagen, Denmark

**Keywords:** transcriptomic, big data, computational model, fibroblast, cellular senescence, temporal profile

## Abstract

Cellular senescence is a diverse phenotype characterised by permanent cell cycle arrest and an associated secretory phenotype (SASP) which includes inflammatory cytokines. Typically, senescent cells are removed by the immune system, but this process becomes dysregulated with age causing senescent cells to accumulate and induce chronic inflammatory signalling. Identifying senescent cells is challenging due to senescence phenotype heterogeneity, and senotherapy often requires a combinatorial approach. Here we systematically collected 119 transcriptomic datasets related to human fibroblasts, forming an online database describing the relevant variables for each study allowing users to filter for variables and genes of interest. Our own analysis of the database identified 28 genes significantly up- or downregulated across four senescence types (DNA damage induced senescence (DDIS), oncogene induced senescence (OIS), replicative senescence, and bystander induced senescence) compared to proliferating controls. We also found gene expression patterns of conventional senescence markers were highly specific and reliable for different senescence inducers, cell lines, and timepoints. Our comprehensive data supported several observations made in existing studies using single datasets, including stronger p53 signalling in DDIS compared to OIS. However, contrary to some early observations, both p16 and p21 mRNA levels rise quickly, depending on senescence type, and persist for at least 8–11 days. Additionally, little evidence was found to support an initial TGFβ-centric SASP. To support our transcriptomic analysis, we computationally modelled temporal protein changes of select core senescence proteins during DDIS and OIS, as well as perform knockdown interventions. We conclude that while universal biomarkers of senescence are difficult to identify, conventional senescence markers follow predictable profiles and construction of a framework for studying senescence could lead to more reproducible data and understanding of senescence heterogeneity.

## Introduction

Multiple studies now suggest that the accumulation of senescent cells is causal in ageing (Childs et al., 2015; Mylonas and O'Loghlen, 2022; van Deursen, 2014; Wlaschek et al., 2021), and their ablation extends healthspan and mean lifespan in rodents (Baker et al., 2016; Baker et al., 2011). Novel senolytic and senostatic drugs are in development (Kim and Kim, 2019; Niedernhofer and Robbins, 2018) with some drugs in clinical trials (Hickson et al., 2019; Justice et al., 2019) which might shortly lead to treatments capable of improving healthspan and extending lifespan in humans. However, the exact nature of senescent cells is often difficult to define, with multiple studies indicating that the most common biomarkers of senescence show different profiles across cell lines, types of senescence inducer, and the timepoint after the initial stimulus (Avelar et al., 2020; Basisty et al., 2020; Casella et al., 2019; Hernandez-Segura et al., 2017; Neri et al., 2021). This makes targeting senescent cells difficult, often requiring combinatorial approaches (Nayeri Rad et al., 2022; Saccon et al., 2021; Xu et al., 2018; Zhu et al., 2015). Although combination therapies can be effective, they also have potential to impact additional molecular networks and their off-target effects can be unpredictable.

To gain a comprehensive understanding of cellular senescence, an integrative, systematic, multi-omic approach is needed. Consequently, we have taken an integrative approach and conducted a transcriptomic systematic review of cellular senescence in human fibroblasts alongside development of a qualitative computational model which simulates protein changes in DNA damage induced senescence (DDIS) and oncogene induced senescence (OIS). We have systematically analysed all transcriptomic data for senescent fibroblasts, meeting pre-specified inclusion criteria, and produced an online database that allows public analysis of the results. Firstly, changes across four types of senescence were compared before analysing temporal changes in key senescence biomarkers in DDIS and OIS, comparing our results to existing single dataset studies. We then examined the literature to build a qualitative protein level model of cellular senescence, using the transcriptomic profiles observed here to aid in the development of the model. For validation of the model, knockdown (KD) interventions of p53 and RelA were performed and compared to data.

This integrated approach has allowed us to address universal biomarkers for senescence, whether findings from individual studies are consistent across the total data, and the mechanisms by which the network of interactants might induce the senescence response. Our results indicated differences in DDIS and OIS for expression of several of the key senescence genes, but profiles were largely consistent with results from individual studies. One exception was the TGF-β-centric SASP identified by Hoare et al. (2016), which does not appear to be replicated at the transcript level.

## Methods

### Systematic review protocol

Three independent systematic searches were conducted and updated to identify all transcriptomic data meeting our inclusion criteria for cellular senescence in human fibroblasts publicly available by 05 October 2023. Datasets were included if they met the following inclusion criteria:

• Unbiased transcriptomic datasets for senescent human fibroblasts. Where senescence was defined exclusively by permanent cell cycle arrest induced by a stimulus in a cell type that would otherwise be proliferating.
• RNA-seq or microarray datasets stored on Gene Expression Omnibus (GEO) (Edgar et al., 2002) or Array Express (Parkinson et al., 2007) by the deadline date of 05 October 2023.
• Data had at least two repeats for all conditions included.

As some datasets meeting the inclusion criteria could not be analysed by the methods described below, they were further excluded if they met the following exclusion criteria:

• Exclusively microRNA or long non-coding RNA datasets.
• Performed at the single cell level.
• Two colour or custom microarrays.
• Data could not be downloaded from GEO or Array Express, nor provided by contact with the corresponding author.

Search terms were developed to include all relevant MeSH terms and text terms that might identify datasets meeting the inclusion criteria. Initial terms were used in combinations on PubMed PubReMiner (Slater, 2014) to identify additional search terms. The search terms selected for GEO and search results of the initial and updated searches are shown in Supplementary Table S1 used in the Advanced Search tool to combine individual searches.

For the smaller Array Express database we searched for “Ageing” OR “Aging” and then manually filtered the results.

As described, manual exclusion was done for both databases in three independent searches firstly on 10 August 2020. At this time the results were compared to an initial non-systematic search of both databases as well as a PubMed search for studies including transcriptomic data, producing a control dataset that ensured the systematic search identified all the studies in the preliminary search. The initial systematic search was then followed by two updated searches, on 06 July 2022 and 05 October 2023. All three searches were done by the same two individuals for two independent searches per search date. After each search, the results were compared to those of the other individual and previous searches to ensure that no studies were missed.

### Database creation

For each study, a comparison matrix was constructed in Microsoft Excel listing all data of interest that could then be combined into a single searchable database. If the data of interest were not available on GEO or Array Express and the datasets had accompanying publications, we checked the papers for any missing data. Key data such as senescence type and cell line were available for all datasets; however, in some cases, the timepoint of senescence induction was not stated in the paper or online databases. As this was a key part of constructing a senescence profile, we then contacted the corresponding author, but we did not do so for any other missing categories.

### Data preparation and analysis

RNA-seq data was downloaded as fastq files from GEO or Array Express. Each file underwent quality check using the fastqcr R package (de Sena Brandine and Smith, 2019) (in R version 3.6.3) and files were compared using the MultiQC BASH command (Ewels et al., 2016). Adapter trimming and removal of low quality read ends was carried out using the Cutadapt tool (Martin, 2011). Once fastq files passed fastqc, or were excluded, they were converted by mapping-based quantification to quant.sf files using Salmon (version 1.1.0) (Patro et al., 2017). The--gcBias--seqBias and--validateMappings options were used to remove additional biases.

For microarrays, series matrix files for selected studies were downloaded from GEOquery and loaded into R using GEOquery (Davis and Meltzer, 2007), converted to esets and labelled with normalisation and processing information provided with the files. Array Express raw data sets were downloaded using ArrayExpress and RMA normalised using affy (Gautier et al., 2004).

Quant.sf files and microarray data underwent differential expression analysis using the R limma package (Ritchie et al., 2015). Data were normalised by cpm or voom commands depending on variance, and plotDensities was used to compare sample curves. Samples with irregular curves not consistent with the rest of the data were removed from further analysis. Log fold change (LogFC) and *p* values were calculated for each comparison defined in the comparison matrix using the eBayes function and combined into a single database for all studies (available on website, see below).

During initial analyses it was noted that the LogFC of some genes were clear outliers. To prevent outliers skewing the analyses, the interquartile range (IQR) was calculated for each gene per timegroup. Outliers were identified as anything less than Q1 (25th percentile) - 1.5*IQR or more than Q3 (75th percentile) + 1.5*IQR. Outliers were removed prior to all analyses presented in this paper.

For some analyses *p* values were inverted (p_i_ value) by the formula in Equation 1. This created a scale that put *p* values for significant upregulation at the opposite end to *p* values for significant downregulation, with non-significant values in the middle.

$$p_{i}=\frac{1}{p}*\frac{LogFC}{\left|LogFC\right|}$$ (1)

Gene set enrichment analysis was carried out using the GSEA command from the ClusterProfiler library (Wu et al., 2021), using the GSEA index h.all.v7.0.symbols.gmt.

We employed the Wilcoxon signed-rank test (which comes in the base R software) for paired sample statistical analysis (Bauer, 1972).

### Online database creation

We transformed the database into a Power BI report. This allows users to access various clusters of the data in easily readable visuals. Data clusters are accessible by button selection and users can sift the data through 13 different filters provided in the report. We used Power BI basic functions and DAX programming language to build the report; specifically, DAX was used to create measures which control the filtering selections. The Power BI report is embedded via an iframe in a Newcastle University research website, available at: https://research.ncl.ac.uk/cellularsenescence. The website holds subsidiary information on the report, project and research team.

### Computational model development

A computational model network was developed for the purpose of qualitatively simulating temporal protein expression changes in DDIS and OIS. A protein network of reactions and interactions in cellular senescence was designed in CellDesigner (Funahashi et al., 2008), informed from extensive literature searching of temporal protein profiles in human fibroblasts (Supplementary Table S2). When there was no available published protein data, transcriptomic data from our systematic database was used as the best available proxy for temporal protein behaviour. A base model composed of reactions, reaction rates and initial parameters was developed using Tellurium (Choi et al., 2018) in a Python 3.6 environment, based on the collected information of the temporal protein profile of senescent human fibroblasts (Supplementary Table S2). Some reactions required Michaelis-Menten kinetics to be applied.

Criteria for the model to meet were devised from commonly accepted senescence phenotypes in the literature as well as from the systematic analysis. KDs (p53 and RelA) were also introduced into the model network to investigate whether senescent KD phenotypes could be successfully recapitulated.

KD phenotype criteria were devised based on the transcriptomic analysis presented here. Meeting of these criteria is how we measured if the protein network could successfully simulate cellular senescence.

To induce senescence, specific inputs were introduced into the base model to induce either DDIS or OIS (Table 1). The inputs were selected and devised based on knowledge from published literature. All expression and time units in the simulations are arbitrary (AU).

**TABLE 1:** Inputs for simulating different cellular states.

Inputs	Cellular states simulated	
	DDIS	OIS
RAS	0.5	5*
DDR	5*	0
kDDRF	14*	0.01
kRASF	0.3*	1*
kDDRFRAS	0.5	5.75*

*Induced once the model had reached equilibrium.

Additionally, an inducible event termed the Notch switch was included in the model network. The Notch switch creates a dynamic change in Notch signalling activity. Activation of the Notch switch results in diminished Notch signalling through the downregulation of NICD (Notch intracellular domain) levels.

Similar to the Notch switch, KDs were simulated through an inducible event. For simulation of a p53 KD, p53 levels and formation of p53 was downregulated. Likewise, for RelA KD simulations NF-κB levels and formation of active NF-κB were downregulated. KDs were induced at the same time as senescence induction, a decision based on the methods described in the p53 and RelA KD studies in the transcriptomic database.

Dynamic sensitivity analysis was performed on the model in both DDIS and OIS conditions at four timepoints (pre-senescence, T20 (Time 20); post-senescence induction, T40; pre-Notch switch, T60; post-Notch switch, T80). We followed the X-method by (Yue et al., 2006) for calculating scaled sensitives to parameter perturbations as ‘events’ at specific simulation time points using SIMBIOLOGY, MATLAB. Results from dynamic sensitivity analysis were as expected, with all proteins appropriately sensitive to senescence inputs and the Notch switch event at the expected timepoints (Supplementary Figure S1).

## Results and discussion

**FIGURE 1:**
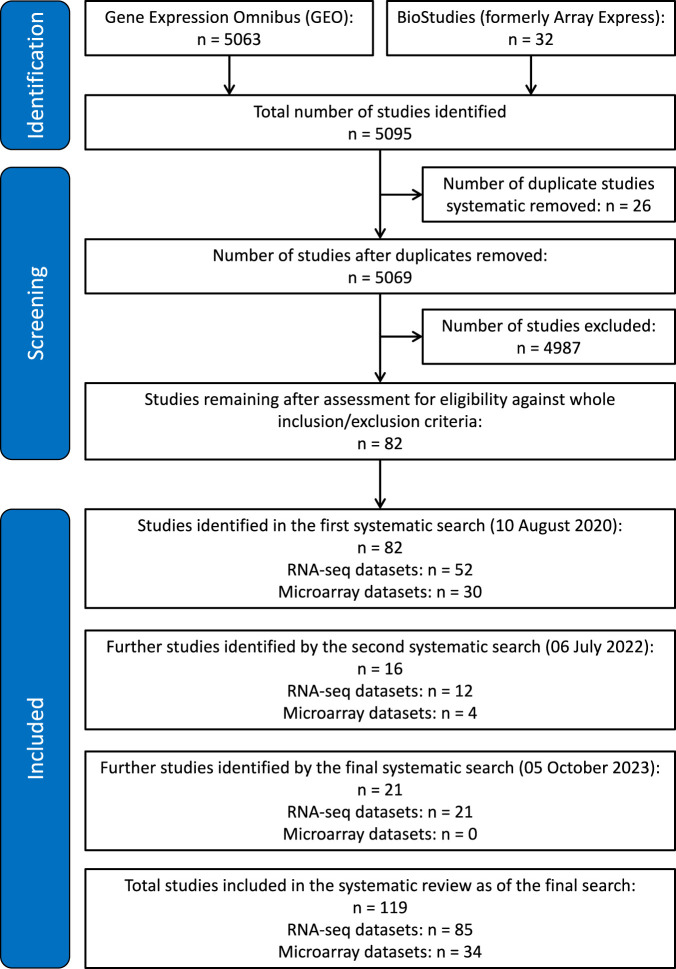
PRISMA flowchart showing identification and exclusion of studies.

The systematic search criteria outlined in the methods initially identified 5063 studies from GEO and 32 studies from Array Express. Of these, 26 were removed as duplicates leaving 5069 datasets for manual analysis against inclusion and exclusion criteria. Of these, 82 were identified as meeting the inclusion criteria and not disqualified by the exclusion criteria. The update searches identified a further 37 studies, as shown in the PRISMA flowchart in Figure 1. A total of 119 studies, including 85 RNA-seq datasets and 34 microarray datasets, were identified in the systematic review.

For each study, comparisons were drawn between sample groups, making a total of 1069 comparisons in the 119 studies. 220 of which were between senescent cells and proliferating controls without treatment or disease. The details of the studies included are shown in Table 2. The main categories, acronyms, and number of comparisons for each are shown in Supplementary Table S3. All data was collated into one large database.

**TABLE 2:** Study data for the 119 studies.

Study ID	Publication	Senescence type	Control type	Cell lines	Timepoints (days, d)	Gene(s) up	Gene(s) down
GSE103938	Aarts et al. (2017)	OIS, OSKM	Prolif	IMR	10d	none	mTOR
GSE94928	Aarts et al. (2017)	OSKM	Prolif	IMR	14d, 20d	none	p21, mTOR
GSE41318	Acosta et al. (2013)	OIS, BYS	Prolif	IMR	7d	none	none
GSE40349	Aksoy et al. (2012)	OIS	Prolif	IMR	7d	none	pRb, E2F7, pRb_E2F7
GSE56293	Alspach et al. (2014)	REP	Prolif	BJ	97PD	none	p38
GSE94395	Baar et al. (2017)	DDIS	Prolif	IMR	10d	none	none
GSE33710	Benhamed et al. (2012)	OIS	Prolif	WI38	7d	none	none
GSE112084	Martínez-Zamudio et al. (2020)	OIS	Prolif, Quiesce	WI38	1d, 2d, 3d, 4d, 6d	none	none
GSE122918	Martínez-Zamudio et al. (2020)	OIS	Prolif	WI38	3d, 6d	none	ETS1, JUN, RELA
GSE143248	Martínez-Zamudio et al. (2020)	REP, OIS	Prolif	WI38	0.5d, 1d, 2d, 3d, 4d, 6d, 11d, 18d, 26d, 33d, 42d, 57d, 88d	none	none
GSE133660	Buj et al. (2019)	dNTP	Prolif	IMR	7d	none	p16
GSE134747	Carvalho et al. (2019)	OIS	Prolif	BJ	1d, 2d, 3d, 5d	GR	RELA
GSE130727	Casella et al. (2019)	DDIS, REP, OIS	Prolif	IMR, WI38	5d, 8d, 10d	none	none
GSE130100	Chan et al. (2020)	OIS	Prolif	BJ	14d	none	none
GSE130099	Chan et al. (2020)	OIS	Prolif	BJ	6d	none	none
GSE19864	Chicas et al. (2010)	OIS	Prolif, Quiesce	IMR	7d	none	pRb, p107, p130
GSE2487	Collado et al. (2005)	OIS	Prolif, Immortal	IMR	3d	SmallT, E6_E7, SmallT_E6_E7	none
E-MTAB-4920	Contrepois et al. (2017)	DDIS	None	WI38	20d	none	H2AJ
GSE76125	Correia-Melo et al. (2016)	DDIS	Prolif	MRC	10d	Parkin	Mitochondrial
E-MTAB-2086	Criscione et al. (2016), Lackner et al. (2014)	REP	Prolif	IMR	30PD, 50PD, 70PD	none	none
GSE109700	De Cecco et al. (2019)	REP	Prolif	LF1	56d, 112d	none	none
GSE70668	Dikovskaya et al. (2015)	OIS	Prolif	IMR	4d	none	none
GSE99028	Dou et al. (2017)	DDIS	Prolif	IMR	7d	none	cGAS
GSE151745	Omer et al. (2020)	DDIS	None	WI38	8d	none	G3BP1
GSE101766	Georgilis et al. (2018)	OIS	Prolif	IMR	6d	none	See table legend†
GSE101750	Georgilis et al. (2018)	OIS	Prolif	IMR	6d	none	PTBP1
GSE101758	Georgilis et al. (2018)	OIS	Prolif	IMR	5d	none	EXOC7, PTBP1
GSE98216	Saint-Germain et al. (2017)	OIS	None	IMR	8d	none	none
GSE127116	Hari et al. (2019)	OIS	Prolif	IMR	5d, 8d	none	LTR2, LTR10
E-MTAB-5403	Hernandez-Segura et al. (2017)	DDIS	Prolif, Quiesce	HCA2	4d, 10d, 20d	none	none
GSE61130	Herranz et al. (2015)	OIS	Prolif	IMR	7d	ZFP36L1	none
GSE122079	Guerrero et al. (2019)	OIS	Prolif	IMR	6d, 7d	none	caspase, ION pump
GSE72407	Gonçalves et al. (2021)	OIS, DDIS	Prolif	IMR	6d, 7d	none	none
GSE42368	NA	DDIS	Prolif	FL2	1d	none	DINO
E-MEXP-2241	Jacobsen et al. (2010)	OIS	Prolif	Tig3	3d	none	miR34a
GSE117444	Mitra et al. (2018)	NA	Prolif, Quiesce	10-5_12 1	7d	none	none
GSE45276	Kennedy et al. (2011)	OIS	Prolif	IMR	7d	none	none
GSE53379	Kirschner et al. (2015)	OIS, DDIS	Prolif, Quiesce, Immortal	IMR	7d	E1A	p53
GSE93535	Lämmermann et al. (2018)	DDIS	Quiesce	HDF161	15d	unknown	unknown
GSE108278	Lau et al. (2019)	OIS	Prolif	IMR	4d, 10d	none	IL1R
GSE75643	Lenain et al. (2017)	OIS	Prolif, Quiesce	Tig3	4d, 10d	SV40smallT	none
GSE134088	NA	DDIS	Prolif	IMR	2d	none	none
GSE94280	Lizardo et al. (2017)	REP	Prolif	BJ	44PD	none	none
GSE42509	Loayza-Puch et al. (2013)	OIS	Prolif, Quiesce	BJ	5d	none	none
GSE131503	Borghesan et al. (2019)	BYS	Prolif	HFF	3d	none	none
GSE63577	Marthandan et al. (2016a)	REP	Prolif	BJ, WI38, IMR, HFF, MRC	26PD, 46PD, 52PD, 57PD, 62PD, 64PD, 72PD, 74PD	none	none
GSE64553	Marthandan et al. (2015)	REP	Prolif	HFF, MRC	22PD, 26PD, 30PD, 34PD,38PD, 42PD, 48PD, 52PD, 58PD, 74PD	none	Complex I
GSE60883	Marthandan et al. (2014)	NA	Prolif	MRC	36PD	none	none
GSE77682	Marthandan et al. (2016b)	DDIS	None	MRC	5d	none	none
E-MTAB-3101	Mellone et al. (2016)	DDIS	Prolif	HFF	7d	TGFb	none
GSE85082	Muniz et al. (2017)	OIS	Prolif	WI38	3d	none	none
GSE28464	Narita et al. (2011)	OIS	Prolif	IMR	4d	none	none
GSE54402	Nelson et al. (2014)	OIS	Prolif	IMR	NA	none	none
GSE42212	Neyret-Kahn et al. (2013)	OIS	Prolif	WI38	5d	none	none
GSE62701	Contrepois et al. (2017)	DDIS	Prolif	WI38	21d	none	H2AJ
GSE120040	Paluvai et al. (2018)	OIS, CR	Prolif	BJ	14d	none	none
GSE128055	Pantazi et al. (2019)	OIS, RiboMature	Prolif	MRC	NA	none	none
GSE24810	Kumari et al. (2021), Rovillain et al. (2011)	OIS	Immortal, Quiesce	HMF3A	7d, 14d	E1A, E7	laminA, p53, E2F, p21
GSE113060	Parry et al. (2018)	OIS	Prolif	IMR	6d	none	HMGA1
GSE37318	Martinez-Zubiaurre et al. (2013)	DDIS	Prolif	CAF	1d	none	none
GSE13330	Pazolli et al. (2009)	REP, DDIS	Quiesce	BJ	4d, 85PD	none	none
GSE60340	Purcell et al. (2014)	REP, DDIS	Prolif, Quiesce, Immortal	LFS_MDAH041	5d, 8d, 18PD, 29PD, 200PD	none	p53
GSE52848	Nelson et al. (2016), Rai et al. (2014)	OIS	Prolif	IMR	8d	none	none
GSE53356	Nelson et al. (2016), Rai et al. (2014)	REP	Prolif	IMR	88PD	none	none
GSE128711	Schade et al. (2019)	DDIS	Prolif	HFF	1d	none	p130, pRb, p130_pRb
GSE105951	Sen et al. (2019)	REP	Prolif	IMR	77PD, 79PD	none	p300, CBP
GSE36640	Shah et al. (2013)	REP	Prolif	IMR	90PD	none	none
GSE19018	NA	REP	Prolif	IMR	30PD, 48PD, 53PD	none	none
GSE23399	Chan et al. (2016)	DDIS	Prolif	CAF	1d, 3d, 7d	none	none
GSE60652	Takebayashi et al. (2015)	OIS	Prolif	IMR	6d	none	pRb
GSE74324	Tasdemir et al. (2016)	OIS	Prolif, Quiesce	IMR	4d, 12d	none	p53, BRD4, RELA, p16_p21, p53_pRb
GSE75207	Tordella et al. (2016)	OIS	Prolif	IMR	7d	none	ARID1B
GSE75291	Tordella et al. (2016)	CR	Prolif	IMR	6d	none	none
GSE132370	Vizioli et al. (2020)	DDIS	Prolif	IMR	10d	none	HDAC
GSE132369	Vizioli et al. (2020)	DDIS	Prolif	IMR	10d	Parkin	mitochondrial
GSE140961	Wakita et al. (2020)	DDIS	None	Tig3	12d	none	BRD4
GSE81368	Wang et al. (2017)	DDIS, REP	Prolif	CAF	NA	none	none
GSE133292	Zhang et al. (2021)	DDIS	Prolif, Quiesce	BJ	12d, 28d	none	p53
GSE98240	Yosef et al. (2017)	DDIS	Prolif	BJ	3d	none	p21
GSE59522	Young et al. (2009)	OIS	Prolif	IMR	0.08d, 0.33d, 2d, 4d,6d, 8d	none	none
GSE98440	Zirkel et al. (2018)	REP	Prolif	IMR	NA	none	none
GSE189789	An et al. (2022)	DDIS	Prolif	WI38	2.5d	none	none
GSE175686	Barnes et al. (2022)	DDIS	Prolif	BJ	1d	none	none
GSE153921	Innes et al. (2021)	OIS	Prolif	IMR	5d	none	XPO7
GSE168994	Lee et al. (2021)	DDIS	Prolif	IMR	10d	none	none
GSE156648	Leon et al. (2021)	OIS	Prolif	IMR	4d	DOT1L	DOT1L
GSE139563	López-Antona et al. (2022)	BYS	Prolif	IMR	4d, 7d, 10d	none	none
E-MTAB-9714	Mangelinck et al. (2020)	DDIS	Prolif	WI38	9d	none	H2AJ
GSE144752	Montes et al. (2021)	OIS	Prolif	BJ	3d	none	MIR31HG, YBX1
GSE112530	Park et al. (2021)	REP, DDIS, OIS, NBIS	Prolif	HDF	8d, 10d, 12d	none	none
GSE77074	NA	DDIS	Prolif	HDF	5d	none	none
GSE124609	Sabath et al. (2020)	REP	Prolif	WI38	4d	none	none
GSE141991	Liu et al. (2021)	OIS	Prolif	IMR	7d	none	METTL14
GSE200479	Zhu et al. (2022)	OIS	Prolif	BJ	14d	none	CBS, p53, NF1
GSE169037	Anerillas et al. (2022a)	DDIS, apoptosis	Prolif, apoptosis	IMR	2d	none	none
GSE145650	Gonçalves et al. (2021)	OIS	Prolif	IMR	6d	none	COX2
GSE178115	Yang et al. (2022)	REP	Prolif	HDF	3d, 8d, 15d	none	none
GSE72404	Hoare et al. (2016)	OIS, NIS, RNIS	Prolif	IMR	6d	none	none
GSE102537	Debès et al. (2023)	REP	Prolif	IMR	NA	none	none
GSE155903	Guerrero et al. (2022)	OIS	Prolif	IMR	6d	none	none
GSE179465	NA	MitoSkip	Prolif	HCA2	21d	none	none
GSE180406	Rey-Millet et al. (2023)	REP	Prolif	MRC	PD71, PD75	TRF2	none
GSE184892	Muto et al. (2023)	Trehalose	Prolif	Primary skin fibroblast	1d, 3d, 14d	none	none
GSE190998	Anerillas et al. (2022b)	DDIS	Prolif	WI38	8d	none	NTRK2, BDNF
GSE191055	Hasegawa et al. (2023)	REP	Prolif	HDF	NA	none	none
GSE198396	Wang et al. (2022a)	DDIS	Prolif	HCA2	12d	none	none
GSE210020	Cho et al. (2022)	REP	Prolif	HDF	NA	none	none
GSE212085	Marin et al. (2023)	DDIS	Prolif	IMR	10d	none	none
GSE213993	Rossi et al. (2023)	DDIS	Prolif	WI38	10d	none	BAFF
GSE214409	Wang et al. (2022b)	OIS	Prolif	IMR	7d	WSTF	none
GSE221104	Anerillas et al. (2023)	DDIS	Prolif	WI38	8d	none	none
GSE222676	NA	DDIS	Prolif	IMR	7d	none	ABCA1
GSE235768	Skea et al. (2023)	DDIS, PIIPS	Prolif	BJ, HFL1	3d, 9d, 10d	none	none
GSE175533	Chan et al. (2022)	DDIS, REP	Prolif	WI38	1d, 2d, 3d, 4d, 7d, PD50, PD52, PD53	none	none
GSE224070	McHugh et al. (2023)	DDIS, OIS	Prolif	IMR	10d	none	COPB2
GSE224071	McHugh et al. (2023)	OIS	Prolif	IMR	10d	none	none
GSE225095	Papaspyropoulos et al. (2023)	DDIS	Prolif	IMR	7d	none	none
GSE234417	Gulen et al. (2023)	DDIS	Prolif	WI38	20d	none	none
GSE196610	Victorelli et al. (2023)	DDIS	Prolif	IMR, MRC5	10d	mtDNA	BAK, BAX

Prolif, proliferating cells; Quiesce, quiescent cells; Immortal, immortalised cells; PD, population doublings; CAF, cancer associated fibroblast; BYS, bystander induced senescence; DDIS, DNA damage induced senescence; OIS, oncogene induced senescence; REP, replicative senescence; CR, chromatin remodelling induced senescence; NBIS, nuclear breakdown induced senescence; NIS, Notch induced senescence; RNIS, Ras and Notch induced senescence; OSKM, senescence induced as a by-product of pluripotency induction via transcription factors Oct4, Sox2, Klf4 and c-Myc; dNTP, depletion of deoxyribonucleotide triphosphates; RiboMature, senescence induced through ribosomal disruption; MitoSkip, senescence induced via mitotic skipping; PIIPS, proteasome inhibition-induced premature senescence; Trehalose, senescence induced though high concentrations of trehalose. †CEBPb, ABCD4, AKR1C1, ALOX5, ASB15, BPIL1, BRD8, C20, CCL23, CTDSPL, DCAMKL3, DUSP11, EMR4, ERCC3, GPRC5D, HSPC182, IFNA17, IL15, IL17RE, ITCH, KCNA5, KCNQ4, LOC399818, LOC51136, MAP3K6, MCFP, NRG1, PEO1, PLCB1, PPP1CB, PROK2, PTBP1, PTPN14, RNF6, SHFM3, SKP1A, TMEM219, UBE2V2, p16, p38, p53, RELA.

Fourteen types of senescence induction were identified from the literature and included in the database: replicative senescence (REP) from telomere erosion (Bodnar et al., 1998); DNA damage induced senescence (DDIS) which can be induced in a number of ways including UV and ionising irradiation or the use of compounds such as etoposide, leading to constitutive activation of the DNA damage response (DDR) and the expression of cell cycle inhibitors; oncogene induced senescence (OIS) occurring through the aberrant activation of oncogenes such as RAS or BRAF; secondary paracrine bystander senescence (BYS) in which neighbouring cells become senescent in response to secreted factors from primary senescent cells; senescence induced through chromatin remodelling (CR); the breakdown of the nuclear barrier leading to nuclear barrier induced senescence (NBIS); Notch induced senescence (NIS) through ectopic NICD activation as well as Ras and Notch (combined) induced senescence (RNIS) (Hoare et al., 2016); OSKM-induced senescence as a by-product of trying to induce pluripotency; induction of senescence through the disruption of ribosomal function (RiboMature) (Pantazi et al., 2019); depletion of deoxyribonucleotide triphosphates (dNTP) induced senescence (Buj et al., 2019); senescence induction through mitotic skipping through the inhibition of CDK1 and MDM2 (MitoSkip) (Johmura et al., 2014); inhibition of the proteasome with drugs such as bortezomib, leading to proteasome inhibition-induced premature senescence (PIIPS) (Skea et al., 2023); highly concentrated treatment with trehalose (Trehalose) (Muto et al., 2023). Control cells could be proliferating or quiescent, and some lines were immortalised or treated with agents that immortalised them as part of the study. Twenty studies compared senescent cells to cells immortalised primarily through hTERT activation, although one study used immortalised cells with p53 knockout (Purcell et al., 2014).

Some comparisons included treatments such as sh/siRNAs against genes designed to observe their effects on senescence, while others used cells from patients with diseases such as breast cancer (Chan et al., 2016), non-small-cell lung cancer (Martinez-Zubiaurre et al., 2013), and Li-Fraumeni syndrome; an inherited syndrome causing vulnerability to rare cancers (Malkin, 1993), here due to mutation of p53 (Purcell et al., 2014). Overexpression of the mitochondrial related gene Parkin (Correia-Melo et al., 2016; Victorelli et al., 2023; Vizioli et al., 2020) also affected gene expression. Another study looked at senescent cells treated with compound “1201,” an alcoholic extract from the plant *Solidago alpestris,* that had unknown effects on gene expression (Lämmermann et al., 2018).

The database, named SenOmic, hosts all the processed transcriptomic data identified in the systematic analysis. To make SenOmic widely accessible, we created a website allowing users to filter for multiple variables to find studies and genes of interest. Users can identify all study or gene data meeting these criteria. For example, comparisons that meet multiple criteria such as ‘OIS in skin fibroblasts with p53 inhibition *versus* proliferating controls’ can be made using the online database available at: https://research.ncl.ac.uk/cellularsenescence. The median LogFC and *p* values can also be calculated, and the data can be downloaded for further analysis. The website comes with an “About” page that explains further details.

### Comparison of senescence profiles and biomarker identification

**FIGURE 2:**
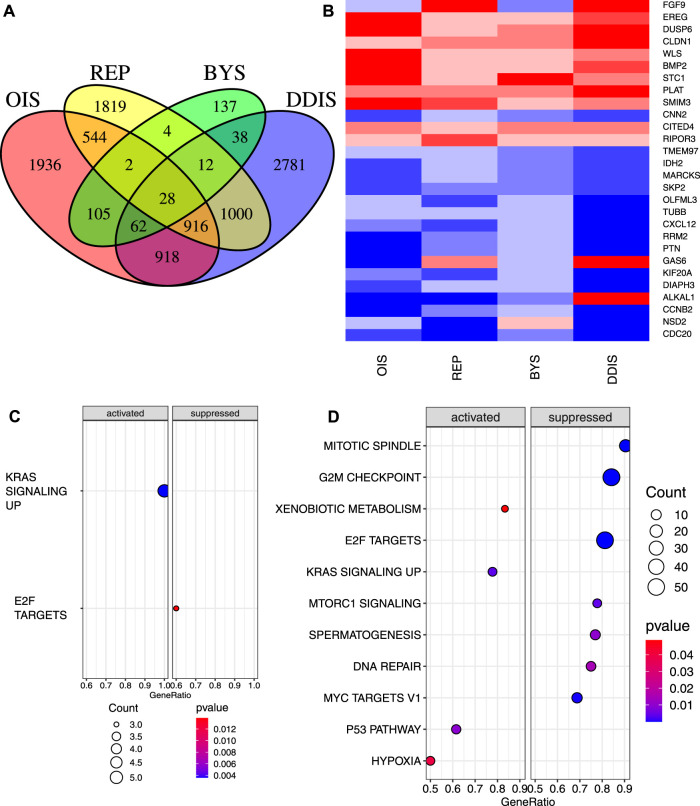
Significant genes and pathways across four types of senescence compared to proliferating control cells. **(A)** Venn diagram of genes with median p_i_ value that is significant across OIS, REP, BYS and DDIS. **(B)** Heatmap of the 28 genes that were significant for all four senescence inducers. Upregulated, red; downregulated, blue. **(C, D)** Dot plot of pathways from GSEA of significantly activated and suppressed pathways identified in **(C)** the 28 genes common in OIS, REP, BYS and DDIS and **(D)** 916 genes common in OIS, REP and DDIS. *p*-value refers to the significance of the overrepresentation of the pathway and count reflects the number of genes associated with the pathway. OIS, oncogene induced senescence; REP, replicative senescence; BYS, bystander induced senescence; DDIS, DNA damage induced senescence; GSEA, gene set enrichment analysis.

In our initial analysis, we included only the 220 comparisons between senescent cells and proliferating controls without genetic abnormalities or treated with agents that altered gene expression (outside of genes such as RAS, RAF and RCC1 used to induce senescence). Of the 220 comparisons of senescence vs. proliferating controls, 196 of them involved REP, DDIS, OIS, or BYS. We therefore compared these four types of senescence to see which genes were commonly significantly different to proliferating cells. For this calculation we used the inverted *p*-value (p_i_ value) (see Methods) so that genes that showed repeated significant change including both increases and decreases compared to proliferating cells were excluded from the set of genes that showed significant change in a consistent direction. The median value was calculated for each gene for each senescence inducer, and a Venn diagram was plotted showing which genes had significant values for which groups (Figure 2A). 28 genes were identified as significant for all four types of senescence; 14 genes consistently downregulated, 10 genes consistently upregulated and 4 genes with regulation dependent on senescence type (Figure 2B). Gene set enrichment analysis (GSEA) of these 28 genes revealed significant suppression of E2F targets and significant activation of genes upregulated by KRAS signalling (Figure 2C). The identification of only two pathways enriched across the four types of senescence mainly reflected that BYS cells had fewer significant changes across all BYS studies (a total of 388 significant genes compared to DDIS, OIS and REP which all identify over 4000 significant genes). There were 916 genes showing consistent and significant changes for OIS, DDIS, and REP. As might be expected, GSEA of the 916 genes common to OIS, DDIS and REP showed significant suppression of the mitotic spindle, G2M checkpoint, E2F targets, and DNA repair (Figure 2D), all of which suggest inhibition of the cell cycle and the DDR. There was additionally significant inhibition of MTORC1 signalling, spermatogenesis, and MYC targets, while there was significant activation of xenobiotic metabolism, KRAS signalling, p53 pathway and hypoxia pathways. Suppression of MTORC1 signalling is interesting as there is a large focus on rapalogues and how inhibition of mTOR can extend lifespan (Weichhart, 2018). However, some studies have found that mTOR signalling is required for senescence (for example, PTEN-loss-induced cellular senescence (Jung et al., 2019)), and that inhibition of mTOR with rapamycin can delay senescence progression (Demidenko and Blagosklonny, 2008; Xu et al., 2014).

Similarly, GSEA for the 918 genes showing significance for both OIS and DDIS indicated these pathways plus the activation of TNFα signalling via NF-κB and the suppression of genes involved in downregulating the UV response (Supplementary Figure S2A). When performing GSEA with a less stringent *p*-value (*p* < 0.1), the 1000 genes significant to only DDIS and REP and the 137 genes significant for just BYS identified no enriched pathways. GSEA of the 388 genes identified as significantly expressed in BYS identified only 3 pathways as significantly activated–the inflammatory response, TNFA signalling via NF-κB, and coagulation (Supplementary Figure S2B), none of which are related to cell cycle arrest or DNA damage. Identification of the inflammatory response and TNFα signalling via NF-κB, however, does support existing literature which demonstrate the inflammatory SASP and NF-κB as being causal in inducing bystander senescence (Acosta et al., 2013; Nelson et al., 2018). The analysis strongly suggested that the common changes in expression for senescent cells, with the exception of BYS cells, is the suppression of the cell cycle and DDR, with different inducers suppressing different genes within these pathways. The fact that BYS cells are not suppressing these genes is interesting. Of the four senescence inducers, BYS had the fewest studies and comparisons, which increases the impact of outlier studies when calculating the median p_i_ value. Thus, this difference may simply reflect that BYS cells have less data available. However, it may also reflect the difference between primary and secondary senescence which is not yet well understood (Admasu et al., 2021; Teo et al., 2019).

Notably, in their non-systematic review of transcriptomic data, Hernandez-Segura et al. (2017) identified a 55 gene core signature for all types of senescence observed. The analysis included six different fibroblast strains (BJ, IMR90, HFF, MRC5, WI38, and HCA-2) for three different inducers (REP, OIS, and DDIS). None of our 28 genes with significant median p_i_ values were in this 55 gene core signature, and only CNTLN, FAM214B, MEIS1, PLK3 and TSPAN13 were consistent with our 916 genes which excluded BYS. Another study by Casella et al. (2019) produced a 68 gene core signature for cellular senescence (not including BYS), of which only one gene (CLDN1) was consistent with our 28 core gene signature, which was upregulated in both cases. There were sixteen genes consistent with our 916 gene signature excluding BYS: ANP32B, CDCA7L, DHRS7, ELMOD1, HIST1H1A, HIST1H1D, HIST2H2AB, ITPRIPL1, JCAD, KIAA1671, LBR, LRP10, PAM, PARP1, PTMA, and SLC9A7. Only POFUT2 was consistent between the core signatures identified between Casella et al. (2019) and Hernandez-Segura et al. (2017). Notably both studies included non-fibroblast cells, and Hernandez-Segura et al. (2017) only included genes that were also significantly different to quiescent cells. However, the identification of a consistent transcriptional biomarker for senescence is clearly problematic. One possible explanation for the lack of any clear core senescence signature is that the senescence profile may change temporally and our above data spans from day 0 to day 112 post-senescence induction, while Hernandez-Segura et al. (2017) and Casella et al. (2019) only investigate up to day 10 post-senescence induction. To support this systematic analysis, we investigated temporal changes in gene expression of core senescence proteins and developed a qualitative computational model of DDIS and OIS which simulates temporal changes of protein expression as senescence progresses, demonstrating how different stages of senescence are characterised by different signatures.

#### Cellular senescence protein network and phenotypes

**FIGURE 3:**
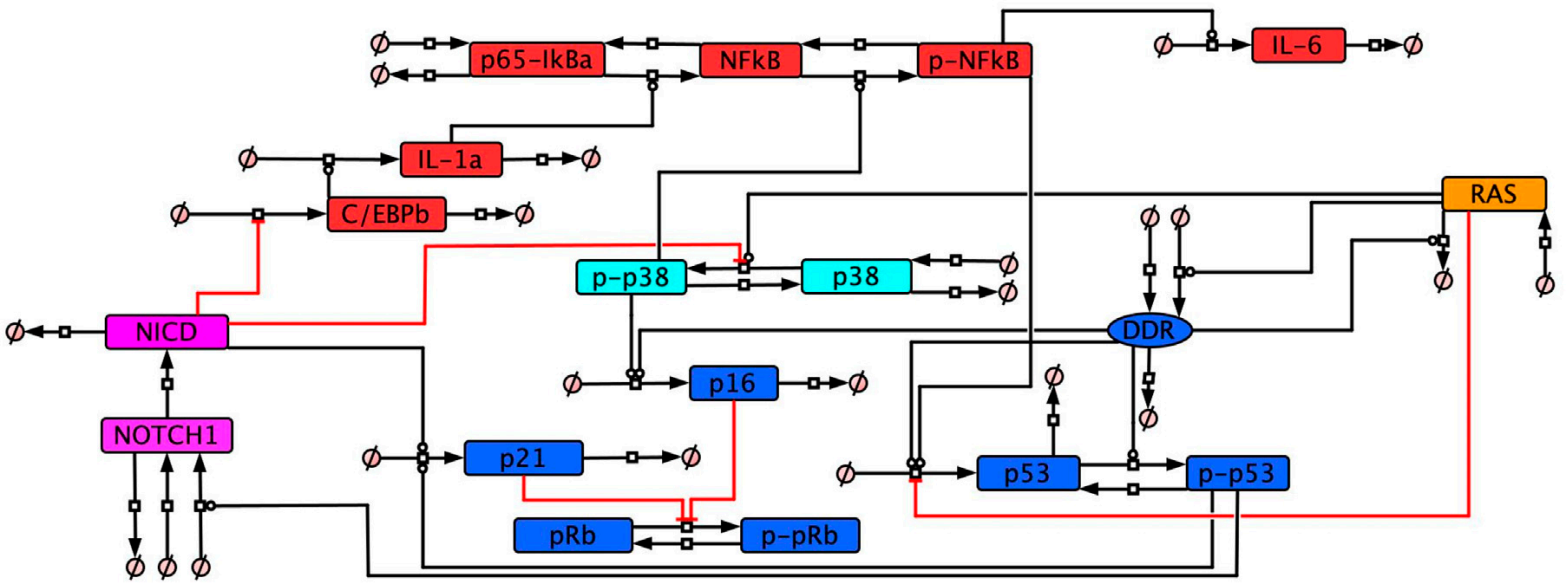
Cellular senescence protein network. The network of interactions between selected proteins recognised as being involved in key aspects of cellular senescence. All interactions in the network have been non-systematically searched in the literature, providing evidence to justify the network (Supplementary Table S2).

The senescence model network was developed based on a non-systematic literature review of protein profiles and our systematic transcriptomic analysis to determine the temporal senescence phenotype. From these combined reviews we developed a total of 12 molecular profiles defining senescence, four of which were related to how senescent cells responded when key network components were knocked down (Table 3). The network is composed of five different interlinking signalling pathways (Figure 3) – cell cycle arrest and DDR related proteins, RAS, p38 signalling, inflammatory signalling, and Notch signalling. The model can be adapted to investigate different dynamic aspects of senescence such as the impact of changes in Notch signalling (Hoare et al., 2016; Teo et al., 2019) or the impact of p53 KD or inhibition (Kumari et al., 2021; Zhang et al., 2021; Zhu et al., 2022).

**TABLE 3:** Phenotypes of cellular senescence.

Senescent cell phenotype	How established is this phenotype at the protein level*	Is this phenotype reflected in the transcriptomic analysis?	Do model simulations match the senescent cell phenotype criteria?
p21 and p16 are still expressed once senescence has been induced for more than 4 days†	3	Y	Y
There is higher p53 activation and p21 expression in DDIS than in OIS	0	Y	Y
There is higher p16 expression in OIS than in DDIS	1	Y	Y
Expression of p16 increases later in DDIS than OIS	0	Y	Y
Changes in Notch signalling activity is temporally associated with a switch in the SASP profile	1	Y	Y
There is a stronger inflammatory phenotype in OIS than in DDIS	2	Y	Y
The inflammatory phenotype begins to be established on days 5 8 after senescence induction††	3	Y	Y
There is more p38 phosphorylation in OIS than in DDIS	1	N	Y
KNOCKDOWN CRITERIA			
Knockdown of p53 results in upregulation of p16 expression	0	Y	Y
Knockdown of p53 results in decreased p21 expression	3	Y	Y
Knockdown of p53 results in upregulation of p38 in DDIS	3	Y	Y
Knockdown of RelA results in decreased p53 signalling	0	Y	Y

*0 denotes no studies can be found related to this phenotype at the protein level; 1 denotes limited published studies support this described phenotype at the protein level; 2 denotes multiple studies support this described phenotype at the protein level; 3 denotes this is a well-established protein phenotype in senescent cells. † 4 days post-senescence induction correlated with arbitrary time 60 in model simulations. †† 5–8 days post-senescence induction correlates with arbitrary time 65-85 in model simulations. Y, yes; N, no; DDIS, DNA damage induced senescence; OIS, oncogene induced senescence; SASP, senescence associated secretory phenotype.

Both the DDR and RAS were included as a source of senescence stimulus. Both DDIS and OIS are known to require activation of the DDR leading to cell cycle arrest (Kumari and Jat, 2021), with hyperactivation of an oncogene such as RAS additionally required for OIS (Liu et al., 2018). In simulations, DDIS is induced by introducing an input which would simulate DNA damage and activation of the DDR to induce cell cycle inhibitor expression and the resultant arrest. While OIS is induced through RAS expression which leads to the activation of p38/p16 with subsequent DDR activation and cell cycle arrest.

The SASP is a key characteristic of senescent cells involved in the spreading of secondary senescence (Acosta et al., 2013; Coppé et al., 2010; Kuilman and Peeper, 2009) and signalling to the immune system for senescent cell removal (Krizhanovsky et al., 2008; Muñoz-Espín and Serrano, 2014). SASP composition can be variable dependent on senescence type and cell type (Childs et al., 2015). However there is also a temporal aspect to the SASP, with some studies observing a shift from a TGFβ-rich secretome in early senescence to an inflammatory-rich secretome in late senescence (Hoare et al., 2016; Ito et al., 2017). The inflammatory SASP is established 5–8 days after senescence induction (Coppé et al., 2010) and is controlled by proteins including p38 (Freund et al., 2011) and NF-κB (Chien et al., 2011). In this study we refer to early senescence as occurring prior to inflammatory SASP induction and late senescence as occurring after induction of the inflammatory SASP.

Studies which have observed this shift from a TGFβ-rich SASP to an inflammatory-rich SASP have observed a dynamic switch in Notch signalling as mediating temporal changes in SASP composition (Hoare et al., 2016; Ito et al., 2017). Notch signalling was therefore included in the model network as a means of mediating a temporal change in SASP composition through an inducible event termed ‘The Notch Switch’. The Notch Switch dynamically changes Notch signalling from active to inactive when induced, allowing for modulation of the SASP.

#### Activity of p53 in senescent cells

Our study and those by Hernandez-Segura et al. (2017) and Casella et al. (2019) indicate that the standard markers of senescence, including those believed to be causal in senescence induction such as p53 and p21, are not reliable biomarkers at the RNA level. Therefore, we looked more deeply at the genes commonly associated with senescence, attempting to identify conditions where they were demonstrably and reliably active or upregulated at the mRNA level.

We first looked at DDR genes thought to play a central role in initiating the senescence response. Double strand breaks or uncapped telomeres activate ATM and ATR followed by downstream CHEK1 and CHEK2 which activate p53 and cause the transcription of p21. Splitting the data into groups by timepoint (0–4 days, 5–7 days, 8–11 days, 12–14 days, and 15+ days), we looked at these damage response genes. Evidence for 12–14 days, excluding REP, is limited to eight comparisons from seven studies, while the 15 + day data is limited to five comparisons from four separate studies only investigating DDIS. The latter was excluded from the graphs due to the limited datapoints. Further research is required at these late time points to complete the temporal profile. REP cells were split into two categories: 0–40 days post-senescence induction and 41+ days post senescence induction. As the vast majority of studies of REP cells did not state the timepoint after induction, these were put in the 0–40 days group under the assumption that waiting 41+ days reflected a deliberate attempt to look at the longterm senescence gene profile and would be noted in the study.

**FIGURE 4:**
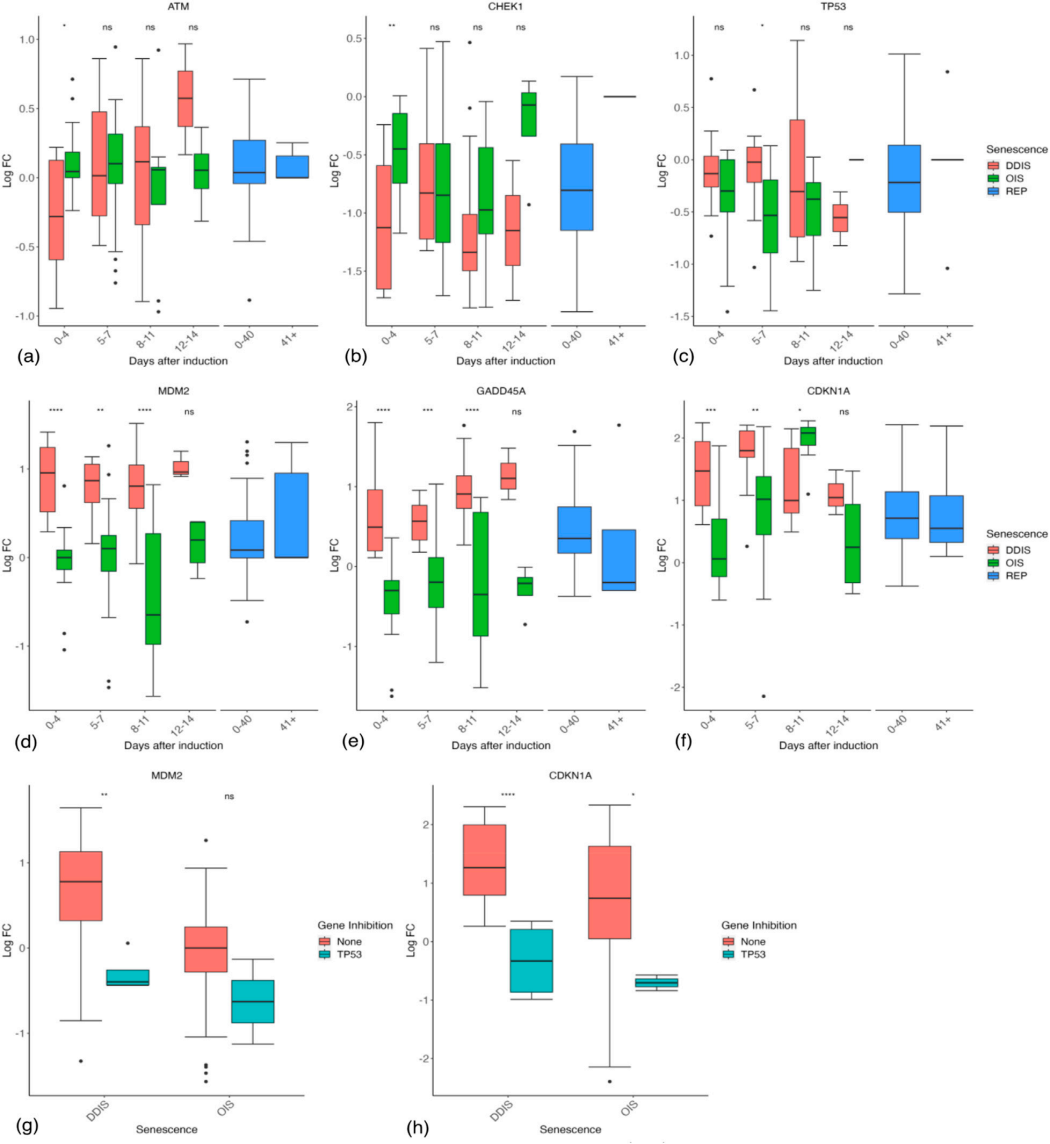
Expression of the damage and p53 response in senescent cells. **(A–F)** Gene expression during the timeline of senescence induction measured in days after the initial stimulus. **(G, H)** Gene expression for different senescence inducers with and without p53 inhibition. Control groups for inhibition include all data for days 1–11. DDIS, DNA damage induced senescence; OIS, oncogene induced senescence; REP, replicative senescence; LogFC, log fold change; *p*-value refers to significance in expression between DDIS and OIS **(A–F)** and expression with and without gene inhibition **(G–H)**, *p-value <0.05; **p-value <0.01; ***p-value <0.001; ****p-value <0.0001.

ATM (Figure 4A) and ATR (Supplementary Figure S3A) mRNAs showed no observable temporal trend in LogFC, in senescence staying around the level of proliferating cells, although ATM expression in DDIS was higher than proliferating cells between days 12–14 post-senescent induction. The same was true for CHEK1 (Figure 4B) and CHEK2 (Supplementary Figure S3B), except that CHEK1 was observably reduced compared to proliferating cells in both DDIS and OIS at least until day 12. CHEK1 and CHEK2 activity is primarily increased at the protein level by phosphorylation by ATM and ATR (Ahn et al., 2000; Jazayeri et al., 2006). A function of both CHEK1 and CHEK2 is to phosphorylate CDC25A, causing its degradation. The mRNA data suggest that CDC25A was decreased compared to proliferating cells (Supplementary Figure S3C), potentially sufficient to induce the S and G2 checkpoints (Falck et al., 2001; Xiao et al., 2003). Additionally, CDC25A expression was significantly lower in DDIS compared to OIS at days 0–4 (*p*-value <0.01) and days 8–11 (*p*-value <0.05). However, the main role of CHEK1/2 is thought to be in the stabilisation of p53 protein (Chehab et al., 2000). p53 signalling is known to be active in senescence (Mijit et al., 2020; Rufini et al., 2013), but this is not reflected in its transcriptional profile, which shows no evidence of an increase in mRNA compared to proliferating cells, and possibly a decrease at some time points (Figure 4C). Not observing a change in p53 mRNA expression likely reflects the pulsatile signalling of p53 (Hunziker et al., 2010; Sun et al., 2011), which is bound and inactivated by MDM2 targeting it for ubiquitin-mediated degradation (Michael and Oren, 2003). Although p53 activity is regulated in large by post-translational modifications and coactivators (Fielder et al., 2017), it is also a short-lived protein, and must in some way be regulated at the transcriptional level; however, the pulses are likely too fast for a single measurement or measurements across multiple days to capture the average level of p53 mRNA compared to control cells (Hunziker et al., 2010; Sun et al., 2011). The level of mouse double minute 2 (MDM2) mRNA, the negative regulator of p53, is increased in DDIS (Figure 4D). Interestingly, expression of MDM2 is significantly higher in DDIS cells compared to OIS cells at 0–4 days (*p*-value <0.0001), 5–7 days (*p*-value <0.01) and 8–11 days (*p*-value <0.0001) post-senescence induction. Although MDM2 inhibits p53, increased expression reflects increased p53 activity, as p53 induces the transcription of MDM2 (Barak et al., 1993). This trend is reinforced by other p53-induced genes such as GADD45A (Kastan et al., 1992) and p21 (CDKN1A) (Figures 4E,F), which are increased across all timepoints in DDIS, while only p21 is notably increased in OIS. Notably, the systematic analysis gives little indication that p53 activity decreases before day 11, with p21, GADD45A, and MDM2 trending to increase at 8–11 days compared with 5–7 days in both OIS and DDIS, typically remaining above proliferating controls at days 12–14 days post-senescence induction.

To confirm the role of p53 in the upregulation of these genes, we looked at the studies which inhibited p53. REP cells were excluded as these cells have no defined time after senescence induction, as was one comparison at day 28, long after p53 signalling is thought to have subsided (Robles and Adami, 1998). As expected, p53 mRNA was observably reduced in p53 inhibition studies (Supplementary Figure S4A). As predicted, the downstream targets of p53, MDM2 and p21 mRNAs, both showed observable reductions in the p53 inhibition group (Figures 4G,H), but interestingly this was not true of GADD45A or B (Supplementary Figure S4B, C). We concluded that although p53 mRNA was not a reliable biomarker of senescent cells (Hernandez-Segura et al., 2017), the combined transcriptional data from all available studies suggest that p53 is highly active in senescent cells up to at least 14 days (Figures 4D–F). However, p53 activity is lower (as measured by p21, MDM2, and GADD45A mRNA levels) in OIS compared to DDIS (Figures 4D–H), suggesting that p53 may play a larger role in DDIS than in OIS.

**FIGURE 5:**
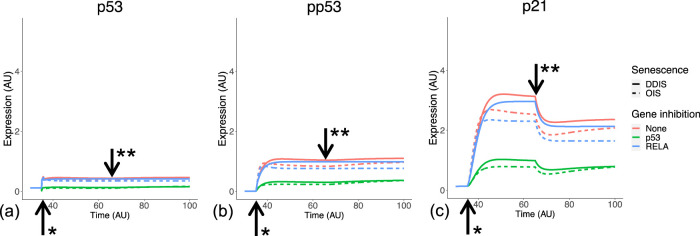
Simulation of p53 signalling dynamics. Simulations show temporal expression of p53 **(A)**, phosphorylated p53 **(B)**, and p21 **(C)**, in DDIS and OIS, and when a p53 or RelA KD is introduced. Units are arbitary (AU). *Senescence and/or KD induced. ** Notch Switch induced. DDIS, DNA damage induced senescence; OIS, oncogene induced senescence.

p53 expression and phosphorylation (pp53) begins at senescence induction, with pp53 increasing as senescence progresses (Freund et al., 2011). This leads to p21 expression and induction of cell cycle arrest. One interesting observation in this transcriptomic analysis is significantly stronger p53 signalling in DDIS compared to OIS up to day 11 (Figures 4D–F), a comparison which has not been made or observed in the literature. When simulating the model, the temporal dynamics of p53, pp53 and p21 meet all criteria devised relating to p53 signalling (Table 3) for both the senescence phenotypes and knockdown phenotypes. There is sustained higher activation of p53 expression in DDIS compared to OIS (Figures 5A,B); p21 is expressed into late senescence (Figure 5C); p53 KD reduced p53 signalling (Figure 5); RelA KD reduces p53 signalling (Figure 5). When the Notch Switch is induced, there is a decrease in the level of p21 expression in both senescence types (Figure 5C), and interestingly we also see a decrease in p21 transcript expression at later timepoints (Figure 4F).

#### OIS and DDIS rely on different mechanisms for arrest

**FIGURE 6:**
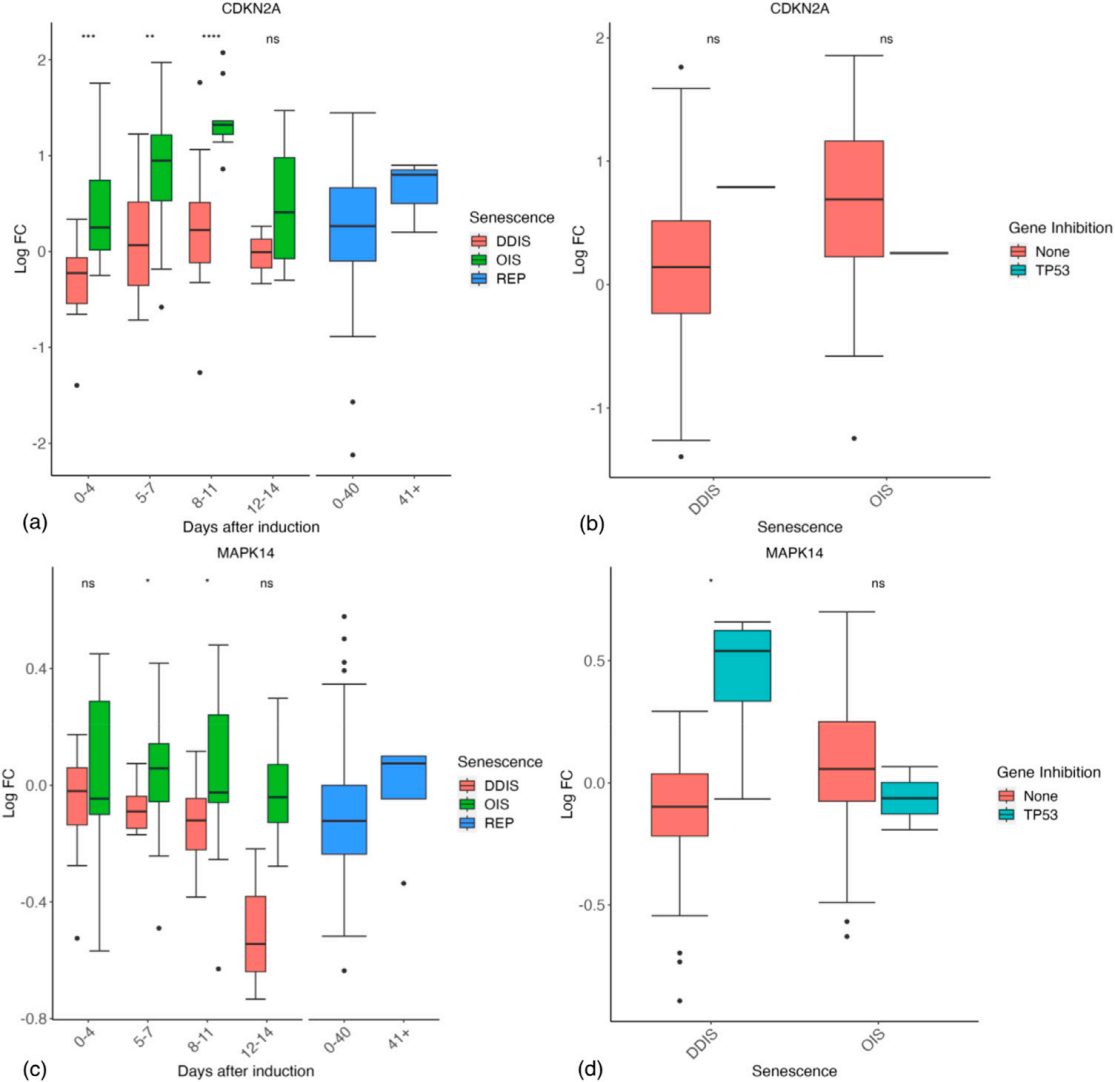
Expression of p16 and p38 genes in senescent cells. **(A, B)** Expression of p16 (CDKN2A) during different timepoints **(A)** and with p53 inhibition **(B)**. **(C, D)** Expression of p38 (MAPK14) during different timepoints **(C)** and with p53 inhibition **(D)**. Control groups for inhibition include all data for days 1–11. DDIS, DNA damage induced senescence; OIS, oncogene induced senescence; REP, replicative senescence; LogFC, log fold change; *p*-value refers to significance in expression between DDIS and OIS **(A, C)** and expression with and without gene inhibition **(B, D)**, *p-value <0.05; **p-value <0.01; ***p-value <0.001; ****p-value <0.0001.

The differences between OIS and DDIS are still being elucidated. DDIS reflects the direct sub-apoptotic chronic induction of the DDR, typically mediated by double strand breaks (DSBs), but OIS need not. Some studies have shown that OIS is bypassed in the absence of the DDR (Bartkova et al., 2006; Di Micco et al., 2006; Mallette et al., 2007), and RAS-induced OIS cells can re-enter the cell cycle if the DDR is inactivated, reflecting that OIS relies on the DSBs induced by the aberrant activation of oncogenes and the resultant hyperproliferation (Di Micco et al., 2006). However, other reports suggest that OIS can be induced independently of the DDR (Alimonti et al., 2010), although still requiring p53 (Wolyniec et al., 2009) or p16 (Bracken et al., 2007). Interestingly, while p21 is significantly higher in DDIS compared to OIS up to day 11 post-senescence induction (Figure 4F), p16 (*CDKN2A*) is significantly higher in OIS compared to DDIS up to day 11 post-senescence induction, and non-significantly higher at days 12–14 (Figure 6A). Upon inhibition of p53 in senescent cells, p16 expression does not significantly change (Figure 6B), suggesting that p16 is independent of p53 signalling (Alcorta et al., 1996).

The activation pathway of p16 is still somewhat controversial. One suggestion is that DNA damage activates p38 (Bulavin et al., 2003; Ito et al., 2006; Iwasa et al., 2003), which then activates p16 (Spallarossa et al., 2010). Our data are consistent with this idea, with p38 (MAPK14) being higher in OIS than DDIS, significantly higher 5–11 days post-senescence induction (*p*-value <0.05) (Figure 6C), and higher in p53 inhibited DDIS cells (Figure 6D). Consistently, Freund et al., 2011 found that p53 inhibited p38 phosphorylation which has implications for the SASP. Several genes showed a stronger response to p53 inhibition in DDIS than in OIS, presumably reflecting that p53 activity is higher in DDIS.

It has been repeatedly suggested that p21 is transient in senescence, required only for induction (He and Sharpless, 2017; Kumari and Jat, 2021). Robles and Adami (1998) showed p21 levels were decreasing by day 8 in DDIS, suggesting that p53 activity peaked around day 4. While this may be true, we saw little evidence of mRNA decline by 8–11 days (Figure 4F), and even at days 12–14 certainly in DDIS expression of p21 mRNA was still higher than in control proliferating cells. Two other frequently cited studies describing transient p21 levels are in REP, and show p21 levels declining over weeks of passaging (Alcorta et al., 1996; Stein et al., 1999). Our systematic analysis indicates p21 levels are still increasing at 8–11 days compared to 5–7 days cells. From this data, p21 seems no more transient than p16, which is generally described to be absent in early senescence and rise slowly over time. Robles and Adami (1998) showed p16 mRNA was no higher than control at day 4 DDIS, slightly increased at day 12 and then peaking at day 30. Stein et al. (1999) indicated that in REP, p16 began its steepest increase after 30 weeks of passaging (compared to 10 weeks for p21). However, systematic analysis indicates that p16 is already increasing in OIS by day 4, and is significantly higher in OIS than DDIS (*p*-value <0.001) (Figure 6A). The rise is less obvious in DDIS, but it does tend to increase temporally up to 8–11 days.

This contrasts p21, which rises at 0–4 days in DDIS but median expression does not increase above 0 LogFC until 5–7 days in OIS. Speculatively, this may reflect that the damage is the primary initiator in DDIS, promptly activating p21, whereas in OIS the damage from hyperproliferation may take longer, while RAS, p38, or other mechanisms independently activate p16. In DDIS, the rise in p16 is slower as reflected by the median LogFC just above zero even by 8–11 days. However, Hoare et al. (2016) show western blots of p16 protein levels increasing by 2 days for both OIS and DDIS. The band at 8 days is observably thicker for OIS than in DDIS, which is consistent with the systematic analysis. Notably, while p38 may activate p16, p38 phosphorylation is increased between 6 and 8 days in OIS (Freund et al., 2011), so it is unlikely to explain the early rise, but if p38 is also activated by RAS signalling (Chen et al., 2000), it may reflect an additional mechanism upregulating p16 in OIS but not DDIS, which might explain the later difference.

Although there is controversy surrounding p16 dynamics in senescence, our analyses suggest p16 expression is maintained into late senescence, significantly more expressed in OIS compared to DDIS, and is expressed in OIS before it is expressed in DDIS (Figure 6A). Our systematic analysis reveals limited change in p38 expression as senescence progresses (Figure 6C), something which is recapitulated at the protein level in the study by Freund et al. (2011) who also demonstrate that while total p38 levels remain similar throughout senescence, phosphorylation levels increase from day 4 to day 6 and continue to increase to at least day 10. As phosphorylation status cannot be explicitly determined at the mRNA level, we are unable to check if this is reflected in the transcriptomic data.

**FIGURE 7:**
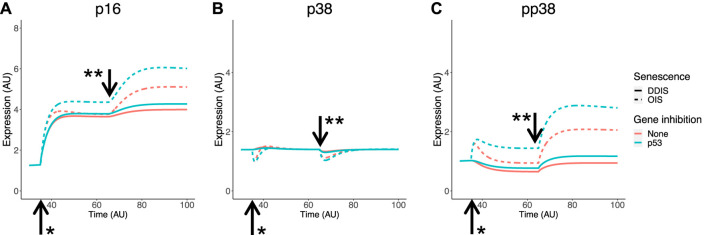
Simulation of p16 and p38 in senescence. Simulations show temporal expression of p16 **(A)**, p38 **(B)**, and phosphoylated p38 **(C)**, in DDIS and OIS, and when a p53 KD is introduced. Units are arbitary (AU). *Senescence and/or KD induced. ** Notch Switch induced. DDIS, DNA damage induced senescence; OIS, oncogene induced senescence.

p16 is involved in cell cycle arrest and is well-known as a senescence marker which has been used for targeted removal of senescent cells in animal models (Baker et al., 2016; Baker et al., 2011). Some studies have observed a link between p38 and p16, with active phosphorylated p38 (pp38) increasing p16 expression (Kwong et al., 2009; Sun et al., 2007), therefore our model network has pp38 contributing to p16 expression. As previously mentioned, phosphorylation status cannot be explicitly investigated at the mRNA level, however it can be in the protein level computational model. Simulation of the senescence model meets all senescence phenotype and KD criteria relating to p16 and p38 outlined in Table 3. Expression of p16 is higher and begins earlier in OIS than in DDIS and is increased when p53 is knocked down (Figure 7A) as expected from our analyses (Figure 6A). One study does find decreased p16 transcript expression when p53 is knocked down (Georgilis et al., 2018) which aligns with our transcriptomic analysis. p38 expression is steady throughout simulations of DDIS and OIS, also consistent with our analyses (Figure 6C) and published protein level studies (Freund et al., 2011), while pp38 increases, particularly in late senescence, and KD of p53 results in increased p38 expression (Figures 7B,C), which is seen at the transcript level (Figures 6B,D).

#### Induction of the SASP

The processes that lead to the induction of the SASP are still uncertain. As discussed by Freund et al. (2011), one possible mechanism is that p38 activates NF-κB. Perhaps the most detailed temporal profile comes from Hoare et al. (2016) who suggested that the initial SASP of OIS and DDIS was a TGFβ-rich secretome, which due to a breakdown in Notch signalling around day 4–5 became an inflammatory secretome.

**FIGURE 8:**
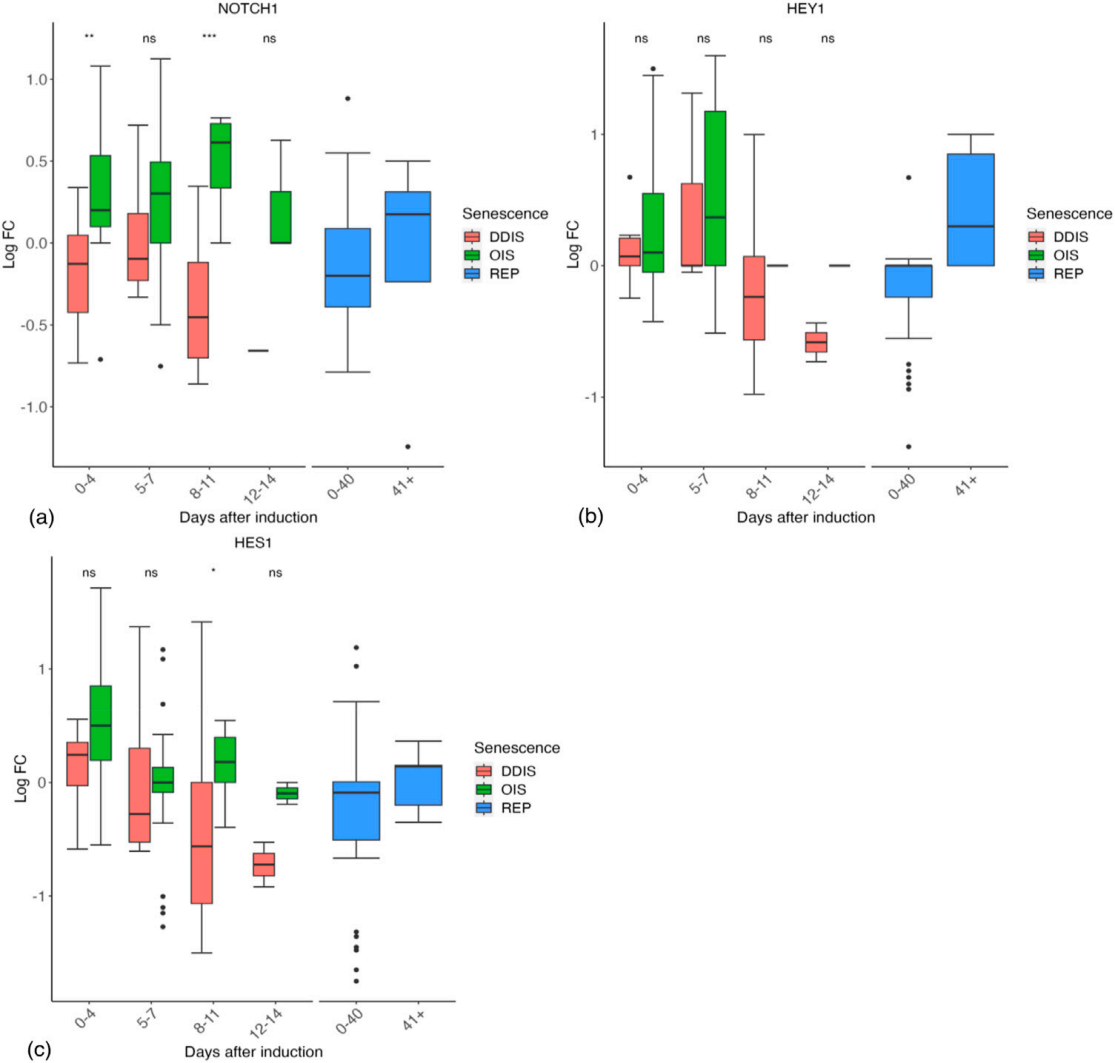
Expression of Notch signalling related genes in senescent cells. **(A–C)** Gene expression during the timeline of senescence induction measured in days after the initial stimulus. DDIS, DNA damage induced senescence; OIS oncogene induced senescence; REP, replicative senescence; LogFC, log fold change; p-value refers to significance in expression between DDIS and OIS, *p-value <0.05; **p-value <0.01; ***p-value <0.001; ****p-value <0.0001.

Notch signalling is a juxtacrine mechanism. When a ligand from one cell binds the Notch receptor of an adjacent cell there are multiple cleavage events which result in the release of the Notch intracellular domain (NICD) which can then translocate into the nucleus and enact Notch targeted gene transcription (Bray, 2006). As Notch signalling is regulated through receptor cleavage and ligand binding, we would not expect to see much change in expression of the NOTCH1 receptor gene. Analysis of NOTCH1 in our database revealed that expression is higher in OIS than in DDIS, being significantly higher at day 0–4 (*p* values <0.01) and 8–11 days (p vales <0.001) (Figure 8A). We then used the database to investigate changes in Notch signalling through expression of two well-known Notch target genes, HES1 and HEY1. It should be noted that NOTCH1 transcriptional activity is still not well understood, even HES1 is not always responsive to NOTCH1 activation (Kopan and Ilagan, 2009; Lee et al., 2007). In our analyses, both HES1 and HEY1 show an increase in median expression compared to control proliferating cells at 0–4 days in DDIS and OIS. Expression stays relatively similar at days 5–7 for HEY1 (Figure 8B) before decreasing at days 8–11, while median HES1 expression decreases from days 0–4 to days 5–7 (Figure 8C). This corresponds with the study by Hoare et al. (2016) who see an initial increase in Notch signalling activity (determinised via the presence of the NICD and HES1) followed by a decrease between days 4–6.

**FIGURE 9:**
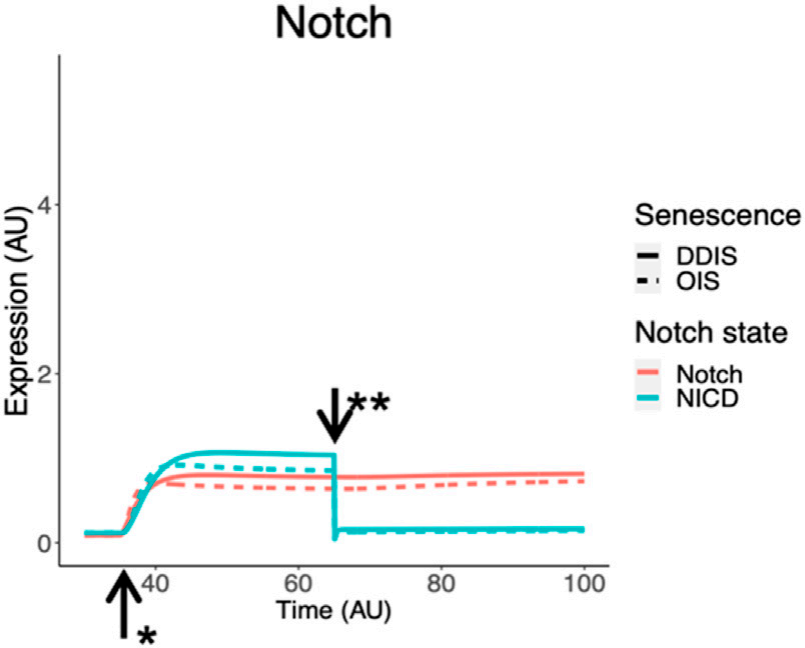
Simulation of Notch signalling dynamics in cellular senescence. Simulations show dynamic temporal changes in Notch signalling from active to inactive in both DDIS and OIS. Units are all arbitrary (AU). *Senescence induced. **Notch Switch induced. DDIS, DNA damaged induced senescence; OIS, oncogene induced senescence.

To model a change in Notch signalling dynamics, we introduced the NOTCH1 receptor and NICD as protein species in the model network. This model is at the single cell level therefore to induce a change in Notch receptor activity we introduced an event termed the ‘Notch Switch’ which results in the downregulation of NICD expression, representing less ligand-receptor activity as less NICD is cleaved from NOTCH1. Simulations demonstrated dynamic changes in Notch signalling temporally. NICD is expressed in early senescence while senescence is being established, indicating active Notch signalling. This is followed by a reduction in NICD and therefore Notch signalling once the Notch switch is activated (Figure 9). Simulation dynamics recapitulate published protein profiles in senescence (Hoare et al., 2016) and reflect our systematic analysis of HES1 transcript expression (Figure 8C), supporting the observation that dynamic Notch signalling is involved in regulation of the SASP.

#### The SASPs of OIS and DDIS are governed by the activity of p38 and p53

NF-κB is essential for the production of the inflammatory SASP (Chien et al., 2011; Freund et al., 2011), and co-suppression with p53 leads to bypass of arrest (Beauséjour et al., 2003). Notably, BJ fibroblasts required only shRNA against RelA to bypass arrest, which the authors concluded may reflect the previously identified less robust senescence program in this cell type (Beauséjour et al., 2003). We compared gene expression for the different cell lines discussed in Supplementary Figure S5 and the results were consistent with a different profile for BJ cells.

**FIGURE 10:**
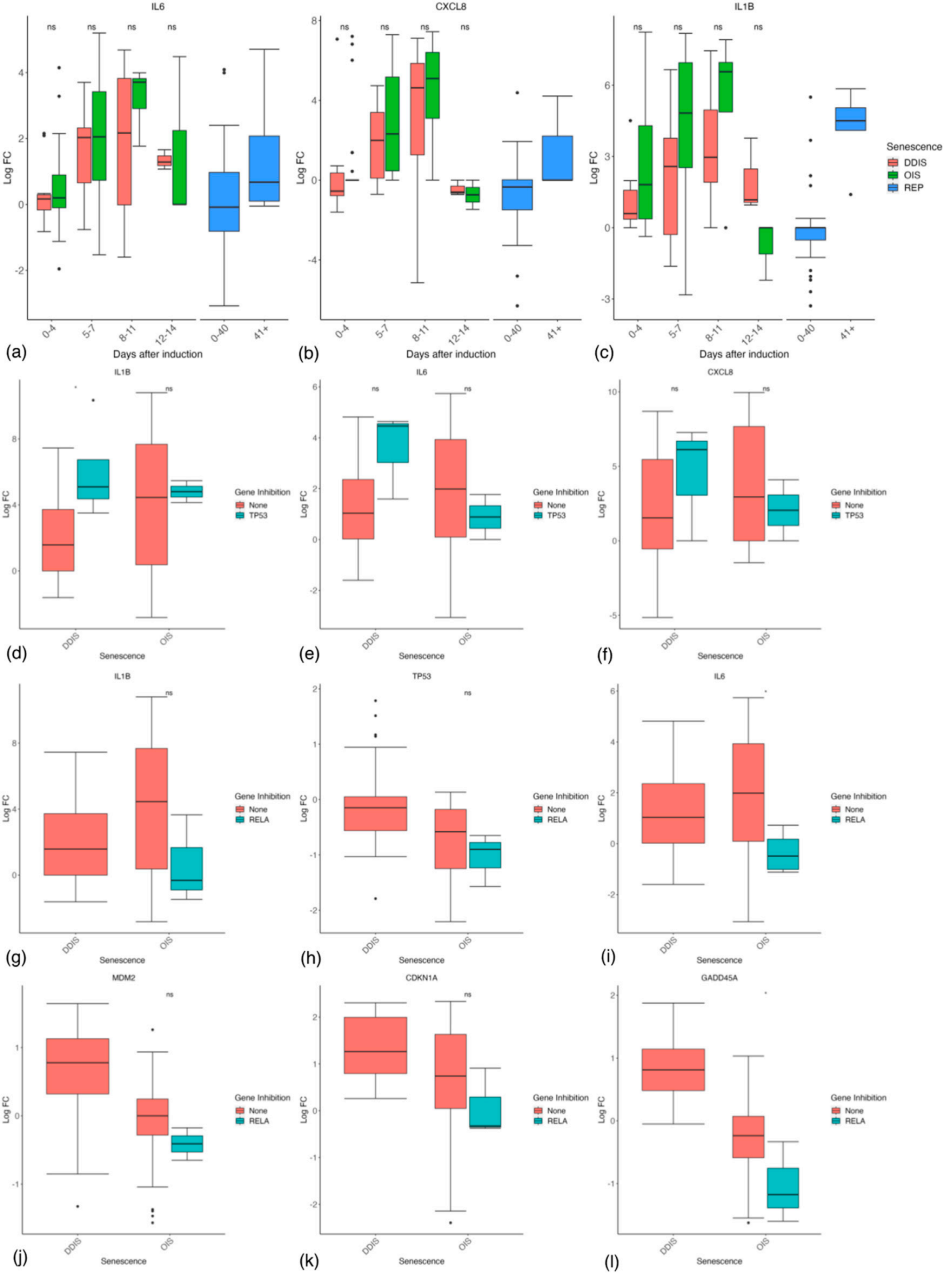
Expression of inflammatory and p53 genes in senescent cells. **(A–C)** Gene expression during the timeline of senescence induction measured in days after the initial stimulus. **(D, E)** Gene expression for different senescence inducers with and without p53 inhibition. **(F–K)** Gene expression for different senescence inducers with and without RelA inhibition. Control groups for inhibition include all data for days 1–11. DDIS, DNA damage induced senescence; OIS, oncogene induced senescence; REP, replicative senescence; LogFC, log fold change; *p*-value refers to significance in expression between DDIS and OIS **(A–C)** and expression with and without gene inhibition **(D–L)**, *p-value <0.05; **p-value <0.01; ***p-value <0.001; ****p-value <0.0001.

Importantly, there is strong evidence of an increased inflammatory response in OIS compared with DDIS, with the main SASP factors including IL-6, IL-8 (CXCL8), and IL-1β all showing higher levels in OIS cells over DDIS at least between 5 and 11 days (Figures 10A–C), although this is not significant at any timepoints. Interestingly, in REP cells the expression of IL-6, IL-8 and IL-1β are all increased at days 40+ compared to 0–40 days whose median expression is around 0 LogFC and therefore similar to proliferating control cells (Figures 10A–C). This shows that while the SASP may be more strongly expressed in OIS, the SASP is still an important mechanism in other types of senescence at later timepoints.

Expression of inflammatory proteins increasing from 5 to 7 days is consistent with the timing of the SASP concluded by others (Coppé et al., 2008; Freund et al., 2011; Hoare et al., 2016). There is also some evidence that p53 is inhibiting the SASP, with trends toward increased IL-1β (*p*-value <0.05), IL-6, and IL-8 in p53 inhibited cells, particularly in DDIS where p53 levels are higher (Figures 10D–F). However, fascinatingly when we looked at the studies where RelA had been inhibited, the results demonstrated the opposite effect. As expected, RelA inhibition showed reduced levels of IL-1β and to a lesser extent IL-6 (Figures 10G,H), suggesting reduced inflammatory signalling. However, both p53 mRNA levels and p53 activity (as represented by MDM2, p21 and GADD45A mRNA levels) were all reduced by RelA inhibition (Figures 10I–L). This strongly suggests that the reduced p53 signalling in OIS is not due to the increased inflammatory signalling, and makes it difficult to explain why p53 activity might peak at 4 days as has been suggested (Robles and Adami, 1998), before upregulation of the SASP. These results are contrary to results by Chien et al. (2011) who investigated the impact of a RelA KD in senescence and found at the protein level when RelA was knocked down, total p53 and p21 expression remained similar to that of control senescent cells.

NF-κB is a major transcriptional regulator of senescence (Oeckinghaus and Ghosh, 2009). In senescence, high level binding of the NF-κB subunit RelA has been shown in complexes at promoters of transcription (Chien et al., 2011) suggesting the active homo/heterodimer in senescence involves RelA. As the most common subunit conformation of NF-κB is p50-RelA (Hoffmann and Baltimore, 2006), we chose to represent NF-κB as a p50-RelA heterodimer in the model network. To be considered a successful representation of cellular senescence, expression of inflammatory proteins must be higher in OIS compared to DDIS, and should not be expressed until senescence has been established at the equivalent of 5–7 days post-senescence induction (Figures 10A–C). Many studies additionally support the activation of the SASP as occurring between 5 and 8 days post senescence induction (Coppé et al., 2008; Freund et al., 2011; Hoare et al., 2016).

**FIGURE 11:**
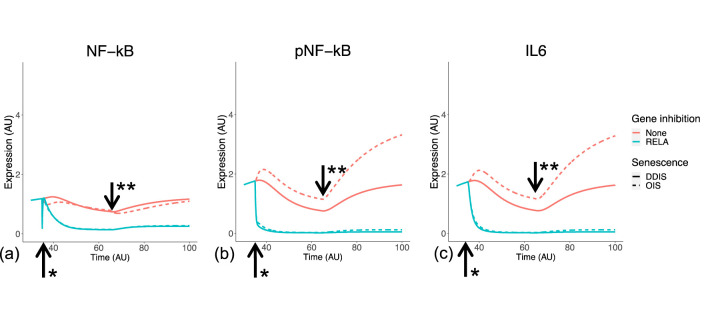
Simulation of the inflammatory SASP in cellular senescence. Simulations show temporal expression of NF-kB **(A)**, phosphorylated NF-kB **(B)**, and IL6 **(C)**, in DDIS and OIS, and when a RelA KD is introduced. Units are arbitary (AU). *Senescence and/or KD induced. **Notch Switch induced. DDIS, DNA damage induced senescence; OIS, oncogene induced senescence.

We simulated SASP induction to begin once the Notch switch is induced at the equivalent of day 5 post senescence induction (Figure 11), producing a stronger inflammatory phenotype in OIS compared to DDIS as pNF-κB and IL-6 expression are both higher (Figures 11B,C). Although NF-κB levels remain similar in OIS and DDIS, there is more pNF-κB in OIS than DDIS, which likely contributes towards the increased inflammatory response observed in OIS in comparison to DDIS, which we also see in our systematic analysis (Figures 10A–C).

Both the systematic analysis and computational model suggest that SASP factors are not delayed in DDIS but are rather reduced in comparison to OIS. For example, median expression of the IL-6 transcript initially increases between days 5 and 7 in both DDIS and OIS; however, it has a larger range of expression in OIS than in DDIS (Figure 10A). The same is true at the protein level as observed in simulations of the senescence model (Figure 11C). While both transcriptomic analyses and model simulations support a stronger expression of SASP proteins in OIS, whether the SASP is primarily contributing towards senescence maintenance or bystander induced senescence is unknown. However, our analyses do find persistent expression of cell cycle arrest proteins such as p53, p21, and p16. Therefore, it could be possible that the inflammatory nature of the SASP is causing further DNA damage to sustain cell cycle arrest. Alternatively, the DNA damage initially caused by the senescence stimulus may not be resolved and could be the cause behind persistent expression of cell cycle arrest proteins.

**FIGURE 12:**
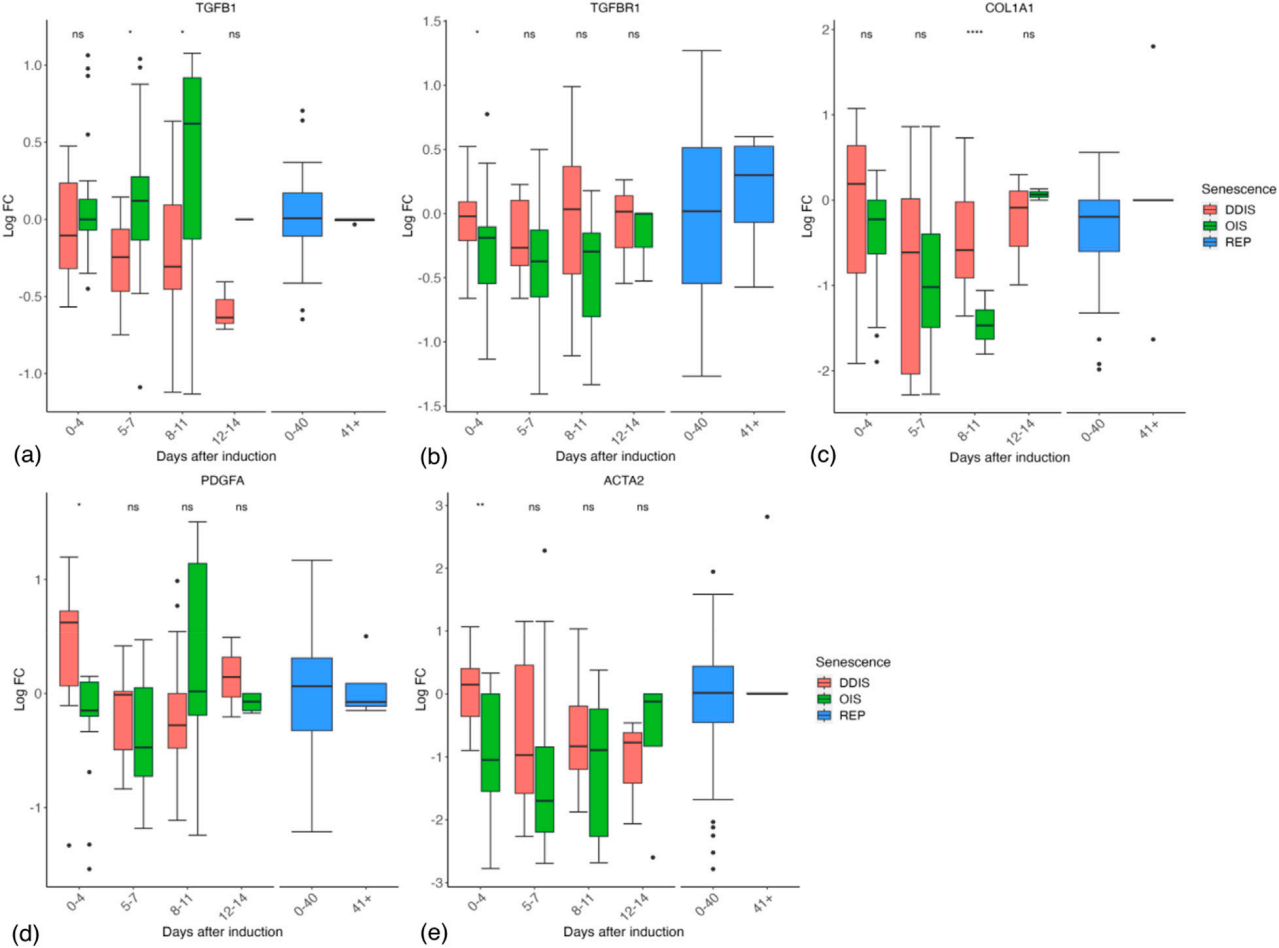
Expression of TGFβ response genes in senescent cells. **(A–E)** Gene expression during the timeline of senescence induction measured in days after the initial stimulus. DDIS, DNA damage induced senescence; OIS, oncogene induced senescence; REP, replicative senescence; IQR, interquartile range; LogFC, log fold change; *p*-value refers to significance in expression between DDIS and OIS, *p-value <0.05; **p-value <0.01; ***p-value <0.001; ****p-value <0.0001.

Contrary to expectations, we could find little evidence of an initial TGFβ-rich secretome at the transcript level in DDIS or OIS as in Hoare et al. (2016). Both TGFB1 and TGFBR1 mRNAs showed no trend toward upregulation at early timepoints followed by a decrease in expression (Figures 12A,B). However, COL1A1, PDGFA, and ACTA2 do tend to decrease in expression from days 0–4 to days 5–7 (Figures 12C–E). Furthermore, the ACTA2 gene encoding the α-SMA protein, a biomarker of myofibroblast development (Wynn and Ramalingam, 2012), which is robustly expressed in response to prolonged TGFβ in both proliferating and senescent cells (Fullard et al., 2024), had reduced expression in OIS at 0–4 days compared to proliferating controls, while DDIS showed no change compared to proliferating cells (Figure 12E). If these observations are correct, the early TGFβ SASP observed by Hoare et al. (2016) may reflect normal function of proliferating fibroblasts that is reduced as Notch signalling declines and the inflammatory SASP activates.

Although we saw no evidence of an initial TGFβ SASP in OIS or DDIS, the data are still far from conclusive, and of course only reflects the senescence profile to the extent it is determined at the transcriptional level. That said, the level of secreted proteins (as would be expected of SASP components) is perhaps well observed at the transcriptional level as the proteins may be quickly secreted and must be replaced by translation of mRNA.

## Conclusion

Here, we have conducted a systematic analysis of all available transcriptomic data for senescent fibroblasts that met pre-specified inclusion criteria and qualitatively modelled temporal protein changes in senescence, including KD interventions.

A total of 12 phenotypic criteria were devised to describe cellular senescence and when a p53 KD or RelA KD was introduced (Table 3), with the model presented here meeting all the criteria. Although this network is not exhaustive of all aspects of cellular senescence, the fact that the model is able to recapitulate different temporal phenotypes found in the literature and in our systematic analyses, supports the strength in the network developed. Dynamic sensitivity analysis was also performed on both DDIS and OIS simulations to determine if proteins species were appropriately sensitive to parameters at different times throughout the senescence process, and it was found that most proteins are appropriately sensitive to direct inputs which induce either senescence or the activation of the Notch switch (Supplementary Figure S1), suggesting that treatment with the same drug but at different doses may result in different outcomes in the transcriptome and therefore at the protein level. In our database, for example, there are nine studies which induce DDIS using etoposide treatment. However, there are three differing doses of etoposide treatment with different treatment regimens depending upon the study design, all of which could lead to changes in the senescence phenotype. Interestingly, this modelling and sensitivity analysis, in addition to the systematic analysis, demonstrate that the temporal profile of a senescent cell is highly sensitive to the stimulus which can result in differing levels of expression.

Additional studies are still required to address how senescence changes over time, particularly at late timepoints. There were multiple variables changed between studies, which likely explains the lack of consistent biomarkers; however, senescence is not a singular defined response, and the senescent phenotype depends on the context of stimulus, cell type, and timepoint among others. As demonstrated with the computational model, the different stimuli for DDIS and OIS results in differing levels of protein changes temporally (particularly in inflammatory SASP components), however there are still common trends between the two types of senescence such as expression of cell cycle inhibitors upon senescence stimulus induction and the SASP becoming active once senescence is established, also observed in the systematic analysis. In conclusion, the results of this systematic analysis suggest that while individual transcripts may not be expressed or repressed with sufficient universality to be used as universal biomarkers of senescence, they do follow predictable profiles depending on the type of senescence and time after induction, which can be modelled computationally. Furthermore, the interplay of different signalling pathways in senescence is a complex temporal process which is yet to be fully understood. Further study should look at cellular senescence as a whole and not just individual pathways disconnected from one another.

The central limitation of this study is its focus on *in vitro* analyses. While senescence has been extensively studied under these conditions, the relevance of the genotypes and phenotypes of *in vitro* senescent cells to those found *in vivo* is still an open question. Particularly how senescence develops and progresses temporally naturally *in vivo* compared to *in vitro*. Even aside from the effects of other cell types and tissues that may alter senescent cells, senescence is such a broad term that we do not yet know whether the cells we are observing *in vitro* are the same types of cell we observe *in vivo*, and many *in vitro* biomarkers do not translate to *in vivo*; for example, the largened, flattened cell morphology observed *in vitro* is rarely observed *in vivo* (González-Gualda et al., 2021). Ultimately, any senotherapeutics identified by *in vitro* analysis will need to be validated *in vivo* in animal models followed by clinical trials. One successful example of this workflow being implemented is with dasatinib and quercetin (D&Q) which were identified through an *in vitro* mechanism-based approach (Zhu et al., 2015). D&Q first proved effective at removing *in vitro* senescent cells, and clinical trials are currently underway investigating D&Q in multiple disease states (Raffaele and Vinciguerra, 2022) including idiopathic pulmonary fibrosis (Justice et al., 2019; Nambiar et al., 2023), Alzheimer’s disease (Gonzales et al., 2023), and diabetic kidney disease. Importantly, preliminary reports in diabetic kidney disease indicate D&Q is successful at eliminating *in vivo* senescent cells (Hickson et al., 2020; Hickson et al., 2019), demonstrating *in vitro* analyses can translate to *in vivo* application.

Another limitation of this study is its methodology of looking for similarities between senescent cells from different cell types, inducers, and conditions, forming a model of the pathways and molecules involved in the senescence process. However, the results of our study and those done previously (Basisty et al., 2020; Casella et al., 2019; Hernandez-Segura et al., 2017; Saul et al., 2022) suggest that senescence is not a static process with universal biomarkers. There is potentially an artificiality in defining all these types of senescence as the same process. However, at least part of the failure to identify universal biomarkers has been the initial focus on biomarkers *per se* rather than understanding the development of senescence. Even if senescence is the same process under all the conditions we look at here, that does not mean it will have the same transcriptional profile at two different points in time. Thus, even the best biomarkers should only ever identify a senescent cell at a particular stage of development. Hence, we have attempted to create a temporal profile of senescence using both bioinformatic and modelling-based analyses. However, while this has several strengths including the use of multiple studies in a combined analysis, it also has the limitation that it is comparing cells from different conditions and studies across the temporal analysis, and even in the combined dataset late timepoints are still lacking.

Importantly, only data from human fibroblasts have been included in the transcriptomic database and protein behaviour in senescence simulations was guided only from studies which used human fibroblasts. This is important as although there is obvious overlap between humans and animal models, there are also notable differences–for example, mice have longer telomeres than humans, shorter lifespans, and cells which escape senescence more easily (Calado and Dumitriu, 2013). The establishment of a common network across all types of senescence could improve the consistency between experimental analysis, which would hopefully lead to more detailed and reproducible results. The database created in this work is hosted publicly online, with an easy to use public-interface to allow further analysis of these 119 combined datasets and expand on the details discussed here. Furthering our understanding of the intricacies and differences in cellular senescence can only increase our chances of producing life-extending senotherapeutic technologies.

## Data Availability

The datasets presented in this study can be found in online repositories. The names of the repository/repositories and accession number(s) can be found in the article/Supplementary Material.
